# Clinical Variability of Classical Ehlers–Danlos Syndrome: A Family with Rare *COL5A1* Variant and Case-Based Review

**DOI:** 10.3390/ijms27177952

**Published:** 2026-09-07

**Authors:** Karina E. Akhiiarova, Ekaterina N. Loginova, Rita R. Kildiyarova, Rita I. Khusainova, Anton V. Tyurin

**Affiliations:** 1 Department of Internal Diseases and Clinical Psychology, Bashkir State Medical University, Ufa 450008, Russia; liciadesu@gmail.com (K.E.A.); ritakh@mail.ru (R.I.K.); 2 Department of Internal Diseases and Family Medicine, Additional Postgraduate Education, Omsk State Medical University, Omsk 644099, Russia; log-ekaterina@yandex.ru; 3 Department of Propaedeutics of Childhood Diseases, N.F. Filatov Clinical Institute of Child Health, I.M. Sechenov First Moscow State Medical University, Moscow 119146, Russia; kildiyarova@mail.ru

**Keywords:** Ehlers–Danlos syndrome, *COL5A1*, WES, NGS, joint hypermobility, skin hyperelasticity, connective tissue diseases, hereditary connective tissue diseases

## Abstract

Ehlers–Danlos syndrome (EDS) comprises a heterogeneous group of inherited connective tissue disorders. The 2017 International Classification of EDS delineates 13 subtypes, which are caused by pathogenic variants in 19 distinct genes encoding various collagen types or proteins involved in collagen metabolism. EDS is characterized by considerable clinical variability, both across EDS subtypes and in terms of phenotypic polymorphism and disease severity within individual subtypes. The present study describes a clinical case of classical-type Ehlers–Danlos syndrome segregating across three generations, illustrating the clinical variability observed within a single family carrying a single rare pathogenic variant, NM_000093.5(*COL5A1*):c.4050dup (p.Gly1351fs). Furthermore, this report clarifies and expands the phenotypic spectrum associated with this specific variant.

## 1. Introduction

Ehlers–Danlos syndrome (EDS) is a heterogeneous group of inherited connective tissue disorders. The 2017 International Classification of EDS delineates 13 subtypes, which are caused by pathogenic variants in 19 distinct genes encoding various collagen types or proteins involved in collagen metabolism [1]. EDS is characterized by considerable clinical variability, both across EDS subtypes and in terms of the diversity of phenotypic manifestations and disease severity within individual subtypes [2].

The most common types of EDS are the classical (cEDS), vascular, and hypermobile subtypes [3]. The diagnosis of cEDS (MIM #130000, #130010) is established on clinical grounds and confirmed by molecular genetic testing through the identification of pathogenic variants in the *COL5A1* and *COL5A2* genes. The major diagnostic criteria include skin hyperextensibility and atrophic scarring, as well as joint hypermobility assessed by the Beighton score, and/or the presence of at least three minor criteria, namely easy bruising, soft doughy skin, skin fragility, molluscoid pseudotumors, subcutaneous spheroids, hernias (or a history thereof), epicanthal folds, complications of joint hypermobility (recurrent sprains and/or ligament ruptures, dislocations/subluxations, chronic pain syndrome, pes planus), and a positive family history [4]. In addition to the major and minor criteria, given the systemic nature of connective tissue pathology, the clinical presentation of patients with cEDS may also include dysfunction of the gastrointestinal, cardiovascular, immune, nervous, and other systems [5]. Of note, within a single family, despite carrying a common pathogenic variant, phenotypic features and the severity of cEDS may vary considerably [6]. Furthermore, in cEDS, similar phenotypes can arise from different molecular genetic defects, posing challenges in the differential diagnosis both among EDS subtypes and within the broader spectrum of heritable hypermobility disorders. Consequently, the study of the molecular genetic landscape of EDS in conjunction with the spectrum of clinical manifestations within affected families remains a pertinent area of investigation.

The aim of the present study was to describe a clinical case of phenotypic trait segregation in a family with classical-type Ehlers–Danlos syndrome across three generations, associated with a rare pathogenic variant in the type V collagen gene.

## 2. Case Presentation

**Figure 1:**
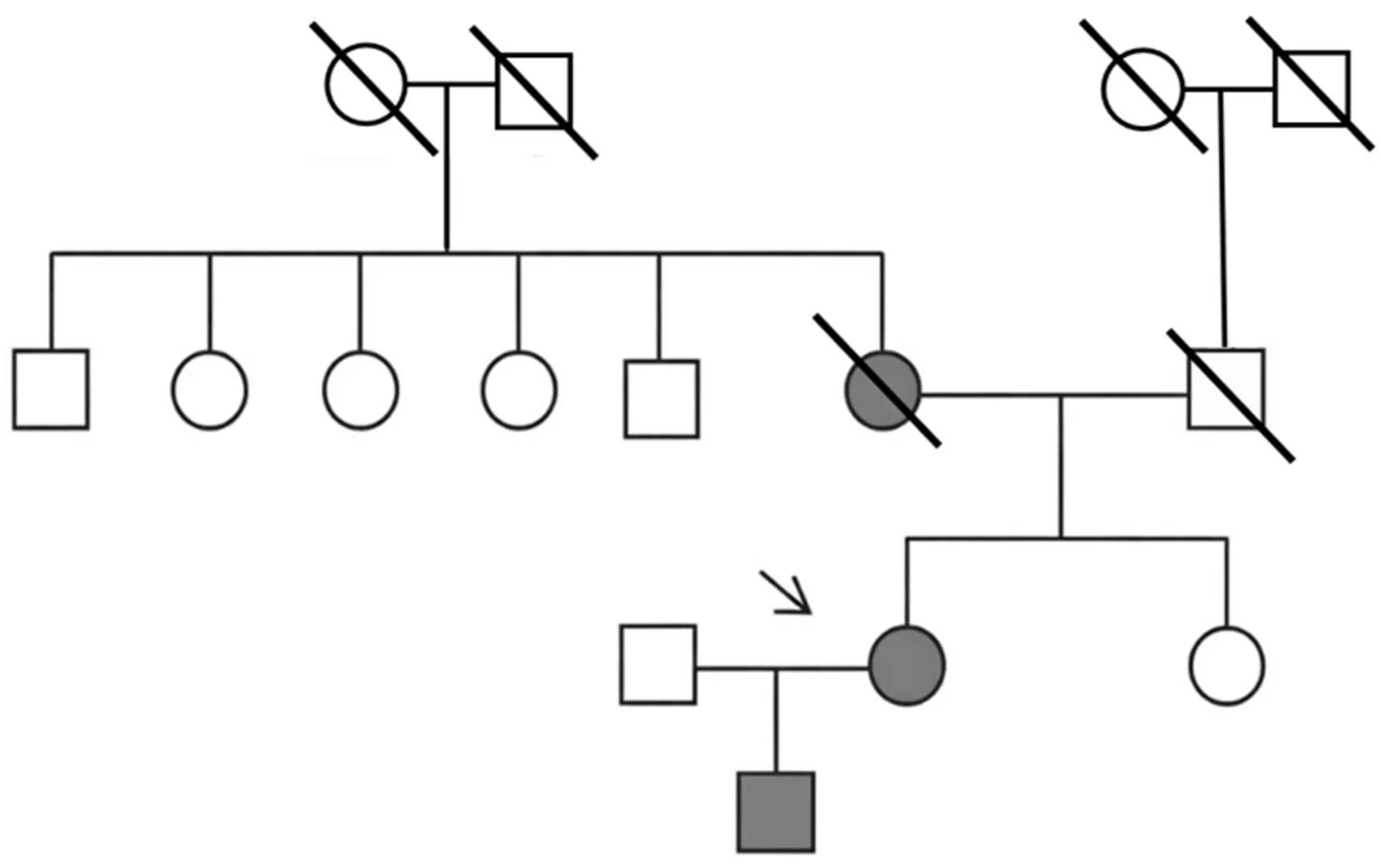
Pedigree of the proband. The proband is indicated by an arrow. Family members with joint hypermobility and skin hyperextensibility are denoted in black.

Medical History: The proband, a 48-year-old female, presented to the Department of Internal Medicine at the Clinic of Bashkir State Medical University with complaints of general weakness, easy fatigability, knee joint pain (rated 3–4 on the Visual Analogue Scale, VAS) without swelling or edema, palpitations, hypotension, thin, fragile, and hyperextensible skin, easy bruising and hematoma formation, particularly over the knee and elbow joints, pectus excavatum (funnel chest deformity), and varicose veins of the lower extremities. The symptoms have been present since childhood: she reported marked joint hypermobility and recurrent shoulder dislocations, chronic pain in the knee and elbow joints, lower extremity varicose veins, and skin fragility. The patient had sought medical attention on multiple occasions for pain management. The patient received symptomatic analgesic therapy with non-steroidal anti-inflammatory drugs (NSAIDs). With advancing age, she noted a decrease in joint hypermobility; however, chronic pain has persisted for many years. Her obstetric history includes two pregnancies, one miscarriage, and one delivery (cesarean section in 2015). The proband’s pedigree is presented in Figure 1.

**Figure 2:**
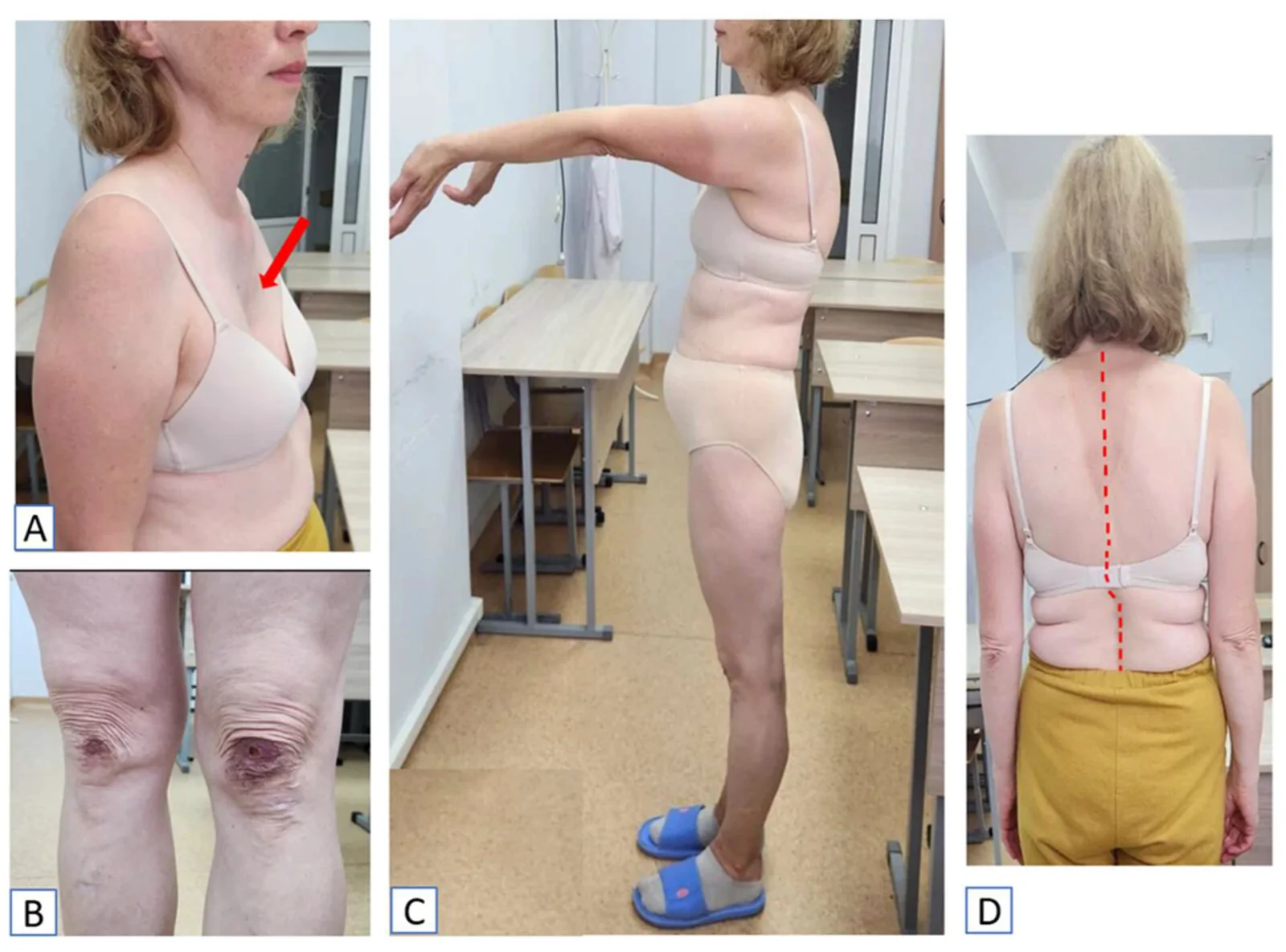
Clinical findings of the proband: (**A**) Pectus excavatum (indicated by arrow); (**B**) skin hyperextensibility with “cigarette-paper” skin; (**C**) joint hypermobility; (**D**) postural abnormality of the scoliotic type (indicated by dotted line).

Physical Examination: The skin was pale pink in color, without pathological rashes, with hematomas over the knee joints and hyperelastic, fragile, “stretched” skin in the region of the knee and elbow joints. Skin hyperextensibility (dorsum of the hand up to 4 cm, elbow up to 2.5 cm, shoulder up to 2 cm) was noted bilaterally. Varicose veins of both lower extremities, subcutaneous spheroids on the lateral and medial surfaces of both feet, atrophic scars on the thighs and abdomen, soft auricles, and pectus excavatum were observed. Joint hypermobility was assessed at 8/9 on the Beighton scale. Blood pressure was 100/60 mmHg, heart rate 67 bpm, and BMI 22.7 kg/m^2^. Bilateral combined pes planus and kyphoscoliotic spinal deformity were also noted. Pain, without swelling or edema, was predominantly localized to the knee joints and the left shoulder joint. Pain in these joints was exacerbated by physical activity and was rated 4/10 on the VAS. Additionally, the patient reported intermittent paresthesia in the upper extremities and a sensation of heaviness in the cervical spine. Photographs of the proband are presented in Figure 2.

**Figure 3:**
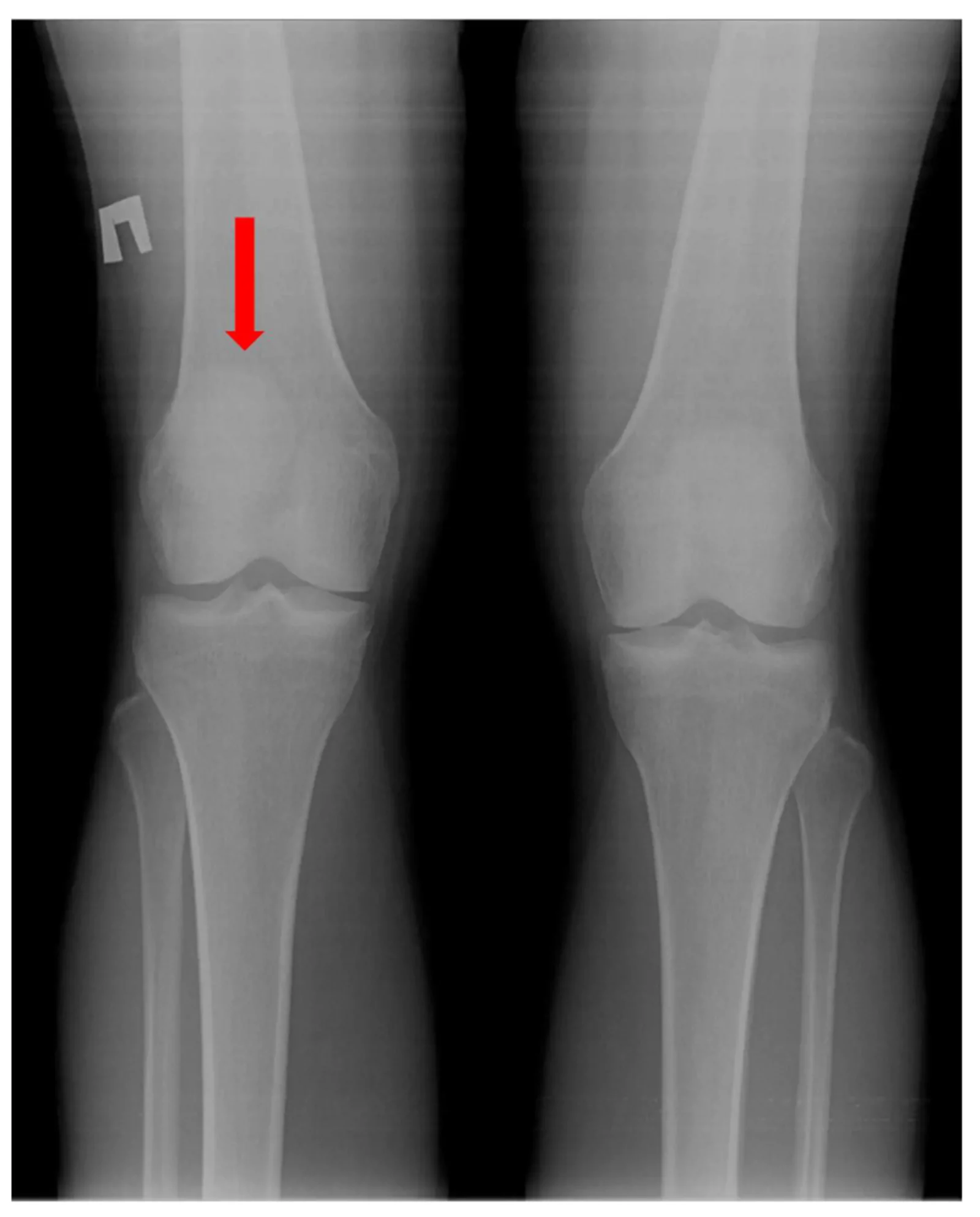
Radiographic imaging of the proband’s knee joints. The knee radiograph demonstrates patella alta on the right side (red arrow).

Radiography of the knee joints was also performed in the proband, as shown in Figure 3, which demonstrates patella alta on the right side.

Routine laboratory investigations revealed no significant deviations from reference values. Electrocardiography demonstrated sinus rhythm with isolated ventricular extrasystoles. Echocardiography showed an ejection fraction of 72%, a left ventricular false tendon (accessory chord), grade I mitral valve prolapse, and mild aneurysmal deformity of the interatrial septum. Abdominal and renal ultrasonography revealed a gallbladder polyp and a left renal cyst. A detailed description of the instrumental findings is provided in the Appendix A.

The proband’s mother presented with the following features: joint hypermobility (7/9 on the Beighton scale), skin hyperextensibility, varicose veins of the lower extremities, and hypertension. She reported complaints of general weakness, pain in the left shoulder joint, episodes of palpitations, blood pressure fluctuations, and easy fatigability. For many years, she had been troubled by bone and joint pain. On physical examination, the skin was of physiological coloration, thin, and hyperextensible, with isolated keloid scars. Blood pressure was 145/95 mmHg, heart rate 77 beats per minute. Her obstetric history included two pregnancies and two deliveries, with no miscarriages. Additional findings included pes planus and mild myopia; she denied any history of surgical interventions. She received therapy for hypercholesterolemia and arterial hypertension. Electrocardiography revealed wandering atrial pacemaker. Echocardiography demonstrated an ejection fraction of 61%, aortic thickening with dilatation at the base, mild aortic regurgitation, biatrial enlargement, a small pericardial effusion, and a right ventricular systolic pressure of 37 mmHg, consistent with pulmonary hypertension. She passed away at the age of 74 years with a diagnosis of ischemic stroke. The results of laboratory and instrumental investigations for the proband’s mother are also presented in the Appendix A.

The proband’s son, aged 11 years, was born at 36 weeks of gestation by cesarean section, with a birth weight of 2380 g, length of 44 cm, and Apgar scores of 6/7 at 1 and 5 min, respectively. His medical history includes obstructive bronchitis, herpetic tonsillitis, and delayed speech development. Hip ultrasonography revealed hip dysplasia. He has also been diagnosed with residual encephalopathy, delayed psychological development, and emotional-volitional disorder syndrome.

**Figure 4:**
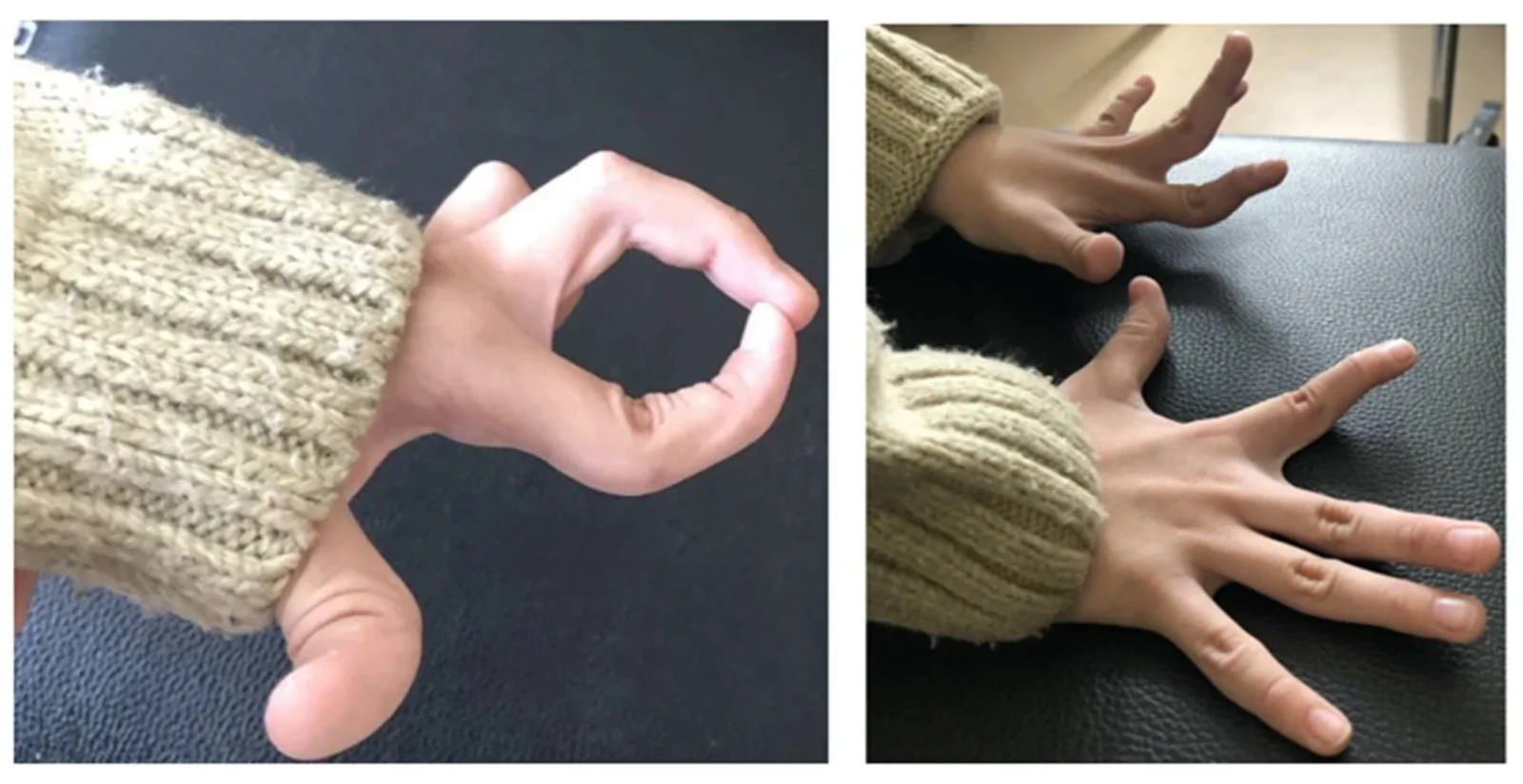
Pronounced joint hypermobility of the finger phalanges in the proband’s son.

Objective examination: The child was lethargic, with pale skin, joint hypermobility of 9/9 on the Beighton scale (Figure 4), keloid scars in the knee region, and skin hyperextensibility. During the consultation, he remained recumbent on the examination couch for most of the time; speech was preserved. Testing for mucopolysaccharidosis types 1, 2, 4, 6, and 3b was negative. At the age of 7 years (2022), he underwent left inguinal hernia repair (Duhamel procedure). Echocardiography revealed a left ventricular false tendon (accessory chord). Doppler ultrasound of the main cerebral arteries with functional tests demonstrated significant compression of both vertebral arteries caudal to the craniovertebral junction and pronounced intracranial venous dyscirculation in the straight sinus. Given the presence of chronic headaches and psychomotor developmental delay, brain magnetic resonance imaging (MRI) was performed at the age of 8 years, which revealed signs of a venous angioma in the left parietal region on native MR tomograms.

A comprehensive summary of the clinical features observed in the proband’s family is presented in Table 1.

The proband and the proband’s son underwent molecular genetic diagnostics by whole-exome sequencing (WES), which identified a previously described nucleotide sequence variant in exon 51, NM_000093.5(*COL5A1*):c.4050dup (p.Gly1351fs). This variant is absent from population databases such as gnomAD. ClinVar contains an entry for this variant (variant ID: 957118). In strict accordance with ACMG criteria, this variant was classified as “Pathogenic” based on the following lines of evidence:

PVS1 (Pathogenic Very Strong): Null variant in a gene where loss of function is a known mechanism of disease. The identified variant is a frameshift indel resulting in a null allele, and loss-of-function is a well-established disease mechanism for this gene.

PM2 (Pathogenic Moderate): The variant is not described in the gnomAD population database.

## 3. Discussion

Classical EDS is the most common monogenic form of EDS. It is typically caused by a deficiency or structural abnormality of type V collagen, a heterotrimeric fibrillar collagen that forms a scaffold upon which type I collagen is deposited for fibril formation (homozygous loss of *COL5A1* expression results in embryonic lethality, with failure of normal collagen fibril assembly) and plays an important role in the assembly and regulation of fibrillogenesis [7]. Inheritance of cEDS is autosomal dominant; more than 90% of patients with the classical type of EDS are carriers of heterozygous pathogenic variants in one of the two type V collagen genes, *COL5A1* or *COL5A2* [8]. The diagnosis of classical EDS is generally straightforward, as most patients present with typical cutaneous manifestations in addition to joint hypermobility; however, molecular genetic verification is required [9] due to the phenotypic overlap with the hypermobile type of EDS.

The present clinical case describes the phenotypic manifestations of classical EDS across three generations of a single family. Upon detailed examination of the observed features, a notable increase in the number of connective tissue pathology signs from generation to generation is evident. Specifically, the proband’s mother presented with joint hypermobility, skin hyperextensibility, atrophic scars, and mitral valve prolapse, while laboratory and instrumental findings were consistent with age-related changes. The proband, in addition to the aforementioned symptoms, exhibited a spectrum of minor cardiac developmental anomalies (left ventricular false tendon, aneurysmal bulging of the interatrial septum, and grade I mitral valve prolapse), pronounced cutaneous manifestations (“cigarette-paper” skin), easy bruising/hematoma formation, and pectus excavatum. The proband’s son, in addition to joint hypermobility, skin hyperextensibility, atrophic scars, and developmental delay, also presented with hip dysplasia, an inguinal hernia (surgically repaired by the age of 11 years), and a verified angioma on MRI. It is also noteworthy that, although the authors describe a familial case of EDS, the frequency and severity of EDS manifestations among all described family members are variable.

The identified variant, NM_000093.5(*COL5A1*):c.4050dup (p.Gly1351fs), has been reported in a limited number of publications. Symoens et al. (2012) described this variant in individuals with Ehlers–Danlos syndrome; however, the associated symptoms and phenotypic features were scarcely detailed [10]. The same variant was also reported by Junkiert-Czarnecka et al. (2013) in a mother–daughter pair [4]. The mother presented with generalized joint hypermobility, velvety skin, easy bruising, joint dislocations, joint pain, and flatfoot, whereas the daughter exhibited joint hypermobility, soft doughy skin, easy bruising, congenital hip dysplasia, scoliosis, and mitral valve prolapse. Notably, as in our clinical case, the child in that report demonstrated a more severe clinical phenotype compared with the mother [4]. Thus, the clinical case described herein represents the most comprehensive report of classical EDS trait segregation across three generations of a single family carrying a single rare pathogenic variant.

Defects in *COL5A1* most commonly represent null alleles, comprising nonsense, frameshift, or splice-site variants that lead to premature termination, as observed in both the proband and her son. Premature termination results in the generation of an unstable transcript that undergoes rapid degradation, leading to an overall reduction in type V collagen levels (i.e., haploinsufficiency) [11,12]. Only a limited number of missense or in-frame exon-skipping variants have been described for *COL5A1* [13]. In the proband and her son, a pathogenic NM_000093.5(*COL5A1*):c.4050dup (p.Gly1351fs) variant was identified, resulting from a duplication of C at nucleotide position 4050. This variant is predicted to introduce a premature stop codon, likely leading to truncation of the encoded protein or its absence due to nonsense-mediated mRNA decay [4,10]. Appendix B presents a hypothetical model of a region of the COL5A1 protein, obtained using AlphaFold, which may be affected by a disruption of nonsense-mediated mRNA degradation. The model is presented for illustrative purposes only and is not evidence of the structural pathogenicity of the variant.

Overall, classical Ehlers–Danlos syndrome is associated with a broad spectrum of complications, including life-threatening ones, such as cardiovascular events—spontaneous arterial ruptures [14,15] and aortic dissection [16,17]—despite the fact that such complications are more characteristic of the vascular type of EDS. Additionally, musculoskeletal pathology is frequently observed, including osteopenia, osteoporosis, recurrent dislocations, and early-onset osteoarthritis [18]. Therefore, to optimize diagnostic and therapeutic strategies, further expansion of knowledge regarding molecular genetic predictors and the clinical manifestations of this disease is required.

## 4. Materials and Methods

### 4.1. Clinical Examination

A clinical examination was conducted on the proband and her family members (the proband’s mother and son) presenting with signs of connective tissue pathology at the Clinic of Bashkir State Medical University, Russia. All participants have provided informed consent in accordance with the standards developed by the World Medical Association (WMA) Declaration of Helsinki “Ethical Principles for Scientific Medical Research Involving Humans.” Written informed consent for the publication of clinical photographs was obtained from all participating individuals. The clinical–genealogical (pedigree) method was employed. The comprehensive clinical evaluation included the assessment of major and minor cEDS criteria, laboratory and instrumental investigations, and genetic testing. Instrumental diagnostic methods comprised electrocardiography, echocardiography, comprehensive ultrasonography of the abdominal organs and kidneys, dual-energy X-ray absorptiometry, and radiography. Laboratory tests included complete blood count, biochemical blood analysis, coagulogram, and urinalysis.

### 4.2. Molecular Genetic Diagnostics

Genomic DNA was extracted from peripheral blood samples using the MagPure Universal DNA Kit (MGI Tech Co., Ltd., Shenzhen, China). Whole-exome libraries were prepared using the Nanodigmbio Exome Capture Kit (Nanodigmbio (Nanjing) Biotechnology Co., Ltd., Nanjing, China) according to the manufacturer’s protocol. Paired-end sequencing (2 × 100 bp) was performed on the DNBSEQ-G400 platform (MGI Tech Co., Ltd., Shenzhen, China). The mean depth of coverage was >100×, with >98% of target bases covered at ≥20×.

Read alignment and post-processing: Raw sequencing reads were aligned to the human reference genome GRCh38 (hg38) using BWA-MEM v0.7.17. Duplicate reads were marked using Picard v2.25.0, and base quality score recalibration (BQSR) as well as local realignment around indels were performed using GATK v4.2.0.0 following best practice guidelines.

Variant calling and filtering: Variant calling was performed using GATK HaplotypeCaller v4.2.0.0 to generate gVCF files per sample. Joint genotyping of all samples was performed using GATK GenotypeGVCFs. Initial hard-filtering was applied to raw variants using the following criteria: read depth (DP) ≥ 10, genotype quality (GQ) ≥ 20, and allele balance (AB) ≥ 0.25 for heterozygous calls. Variants with minor allele frequency (MAF) ≥ 0.01 in gnomAD v3.1.2 were excluded.

Annotation and prioritization: Filtered variants were annotated using ANNOVAR (version 2020-06-08) with reference data from RefSeq, dbSNP v155, ClinVar, and gnomAD. Functional consequence annotations were derived using the Ensembl Variant Effect Predictor (VEP) embedded in ANNOVAR. Prioritization of candidate variants was performed by filtering for rare, protein-altering variants (non-synonymous, splice-site, frameshift, or stop-gain) in genes known to be associated with heritable connective tissue disorders.

Pathogenicity prediction: For retained rare variants, in silico pathogenicity predictions were obtained from SIFT, PolyPhen-2 (HumVar), MutationTaster, and CADD v1.6. A variant was considered to have damaging potential if at least three out of four prediction tools agreed on a deleterious effect. Splice-site effects were additionally assessed using Human Splicing Finder v3.1.

Variant interpretation: All candidate variants were manually inspected using the Integrative Genomics Viewer (IGV v2.11.9) to confirm read alignment, allele balance, and absence of strand bias. Final classification of pathogenicity was performed in accordance with the ACMG/AMP 2015 guidelines (Richards et al., 2015) [19], incorporating population frequency (gnomAD), computational predictions, and segregation data within the pedigree.

The described pipeline was implemented and executed within the Galaxy platform (https://usegalaxy.eu, accessed on 12 January 2026), with additional manual curation steps performed as outlined above.

Prediction of protein structure for the identified variant was performed using the Uniprot.org (https://www.uniprot.org/, accessed on 7 April 2026) database and AlphaFold3 (https://alphafoldserver.com/welcome, accessed on 7 April 2026).

## 5. Conclusions

Thus, to date, this study is one of the few that provides a detailed description of a clinical case of classical Ehlers–Danlos syndrome that segregates across three generations, and demonstrates clinical variability within a single family carrying the rare pathogenic variant NM_000093.5(COL5A1):c.4050dup (p.Gly1351fs), and further clarifies and expands upon the phenotypic characteristics associated with this variant.

### Study Limitations

Our study has a number of limitations. In particular, molecular genetic diagnostics could not be performed for the proband’s mother due to her death; therefore, the authors cautiously suggest, given the presence of clinical features, that she was a carrier of this variant. Another limitation of the study is the lack of Sanger verification.

According to the data obtained from medical records, the severity of the disease increases from generation to generation. However, it is worth noting that all members of the described family were of different ages and did not undergo examinations according to a unified protocol; however, in the future, monitoring with a more consistent methodological approach is planned.

## Figures and Tables

**Table 1:** EDS features identified in the studied family.

Features	Proband’s Mother	Proband	Proband’s Son
Joint hypermobility, score	7	9	9
Skin hyperextensibility	Mild	Severe	Severe
Atrophic striae	−	+	+
Pectus excavatum	-	+	-
Varicose veins of the lower extremities	+	+	-
Skin fragility	+	+	+
Visceral hernias	-	-	+
Minor cardiac developmental anomalies	Aortic valve insufficiency	MVP, left ventricle accessory chord, aneurysmal dilatation of the interatrial septum	MVP, left ventricle accessory chord

MVP—Mitral valve prolapse.

**Figure A1:**
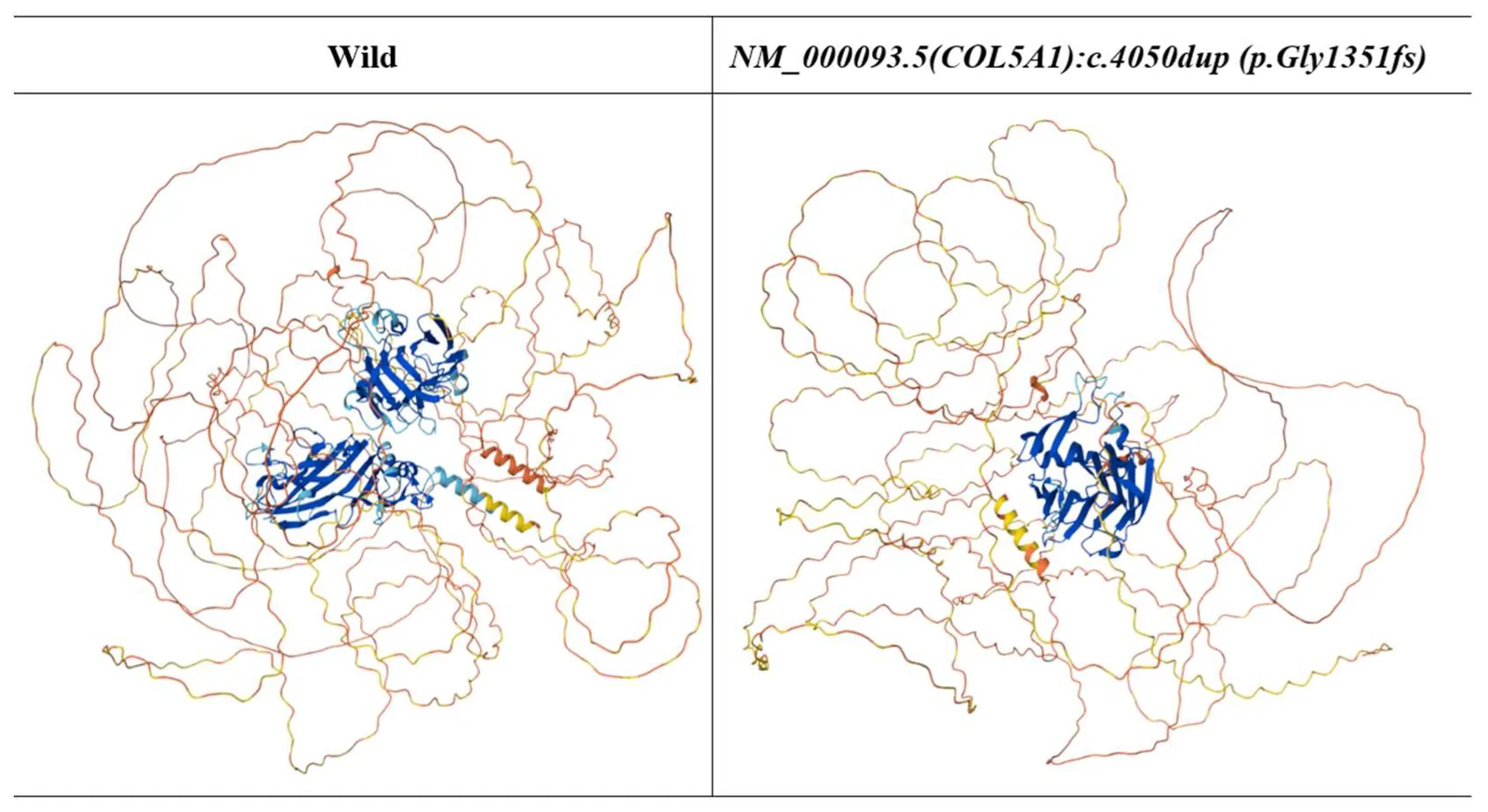
AlphaFold models of wild-type and defective COL5A1 proteins. Hypothetical model of a protein region that would be affected if translation evades NMD. The model is presented for illustrative purposes only and does not serve as proof of the structural pathogenicity of the variant.

## Data Availability

The data that support the findings of this study are available from the corresponding author upon reasonable request.

## Abbreviations

The following abbreviations are used in this manuscript:

EDS	Ehlers–Danlos syndrome
cEDS	Ehlers–Danlos syndrome classical type
VAS	Visual Analogue Scale
WES	Whole-exome sequencing

## Appendix A

**Table A1:** Laboratory and instrumental findings in the proband and the proband’s mother.

Parameter	Proband	Proband’s Mother
Complete Blood Count	Hemoglobin 138 (g/L), Erythrocytes 4.6 (×10^12^/L), Leukocytes 6.2 (×10^9^/L), Platelets 275 (×10^9^/L), ESR 12 (mm/h)	Hemoglobin 132 (g/L), Erythrocytes 4.2 (×10^12^/L), Leukocytes 5.8 (×10^9^/L), Platelets 240 (×10^9^/L), ESR 18 (mm/h)
Biochemical Analysis	Glucose 5.1 (mmol/L), Total protein 74 (g/L), ALT 22 (U/L), AST 20 (U/L), Total bilirubin 12.3 (µmol/L), Creatinine 75 (µmol/L), Urea 5.0 (mmol/L), Total cholesterol 5.2 (mmol/L), HDL 1.5 (mmol/L), LDL 3.0 (mmol/L), Triglycerides 1.2 (mmol/L), Iron 16.5 (mmol/L), Ferritin 65 (ng/mL)	Glucose 5.4 (mmol/L), Total protein 72 (g/L), ALT 19 (U/L), AST 18 (U/L), Total bilirubin 10.8 (µmol/L), Creatinine 68 (µmol/L), Urea 5.8 (mmol/L), Total cholesterol 6.1 (mmol/L), HDL 1.6 (mmol/L), LDL 3.8 (mmol/L), Triglycerides 1.5 (mmol/L), Iron 14.0 (mmol/L), Ferritin 95 (ng/mL)
Coagulogram	APTT 28 (s), Prothrombin time 13.2 (s), INR 0.98, Fibrinogen 3.2 (g/L), Thrombin time 16.5 (s), Antithrombin III 102 (%), D-dimer 220 (ng/mL)	APTT 29 (s), Prothrombin time 13.8 (s), INR 1.05, Fibrinogen 3.8 (g/L), Thrombin time 17.0 (s), Antithrombin III 98 (%), D-dimer, 380 (ng/mL)
Urinalysis	Color Straw-yellow, Clarity Clear, Specific gravity 1.018, pH 6.0, Protein (g/L) Negative, Glucose Negative, Ketone bodies Negative, Leukocytes: not specified; Squamous epithelium: not specified; Mucus: negative; Salts: negative; Bacteria: negative	Color Straw-yellow, Clarity Clear, Specific gravity 1.015, pH 6.5, Protein (g/L) Negative, Glucose Negative, Ketone bodies Negative, Leukocytes: not specified; Squamous epithelium: not specified; Mucus: negative; Salts: negative; Bacteria: negative
**Electrocardiography ** **(ECG)**	Sinus rhythm, heart rate 61 bpm. Horizontal electrical axis. Reduced voltage. Single atrial extrasystole. Impaired repolarization processes in the ventricular myocardium. Impaired intraventricular conduction.	Wandering atrial pacemaker.
**Echocardiography (ECHO)**	Ejection fraction (EF) 72%. Grade I mitral valve prolapse. Left ventricular false tendon (accessory chord). Mild aneurysmal deformity of the interatrial septum.	EF 61%. Aortic wall thickening with dilatation at the base. Mild aortic regurgitation. Biatrial enlargement. Small pericardial effusion. Right ventricular systolic pressure (RVSP) 37 mmHg. Pulmonary hypertension.
**Abdominal and renal ultrasonography**	Gallbladder polyp. Moderate diffuse changes of the pancreas. Left renal cyst.	Hepatic steatosis. Chronic calculous cholecystitis. Renal cysts.
**Dual-energy X-ray absorptiometry (DEXA)**	T-score = 0.6	Not performed/data not available
